# Recently Identified Novel Human Astroviruses in Children with Diarrhea, China

**DOI:** 10.3201/eid1908.121863

**Published:** 2013-08

**Authors:** Yongxia Wang, Yuning Li, Yu Jin, Dan-di Li, Xiaole Li, Zhao-jun Duan

**Affiliations:** The First Hospital of Lanzhou University, Lanzhou, China (Y. Wang, Y. Li);; Medical School of Nanjing University, Nanjing, China (Y. Jin, X. Li);; Department of Viral Diarrhea Institute for Viral Disease Control and Prevention, Beijing, China (D. Li, Z. Duan)

**Keywords:** Astrovirus, diarrhea, China, children, viruses, enteric infections

**To the Editor:** Human astroviruses (HAstVs), first identified in 1975, are now considered an important cause of viral gastroenteritis, predominately infecting children <2 years of age (*1*,*2*). HAstVs are classified into 8 serotypes. A unique astrovirus, MLB1 (AstV-MLB1), recently was discovered in a fecal sample from a child with diarrhea in Australia (*3*); subsequently, at least 6 novel astroviruses have been discovered from fecal samples, including AstV-MLB2, AstV-MLB3, HMO AstV-A/VA2, HMO AstV-C/VA1, HMO AstV-B/VA3, and AstV-VA4 (*4**–**7*). The prevalence of novel astroviruses in China remains unclear.

Fecal specimens were collected during July 2010–June 2011 from 723 children <5 years of age who had acute gastroenteritis. Samples were from all of 295 eligible children brought for care to First Hospital of Lanzhou University (Lanzhou, China) and every fifth eligible child (n = 428) brought for care on 2 days of the week (Tuesday and Thursday) at Nanjing Children’s Hospital (Nanjing, China). The children’s parents provided informed consent. The ethics committees of both hospitals approved the study.

Nucleic acids were extracted from specimens by using the Viral Nucleic Acid Extraction Kit II (Geneaid, Taipei, Taiwan). Adenovirus and caliciviruses were detected by PCR and reverse transcription PCR, respectively (*8*). Rotavirus was detected from fecal samples by ELISA (Oxoid, Cambridge, UK). Primers Mon269/Mon270 detected a region of the capsid gene (449 bp) from classic HAstV-1–8 by reverse transcription PCR (*8*). Additional astrovirus types were detected by using primers SF0073/SF0076, amplifying a 409-bp fragment of the astrovirus gene open reading frame 1b (*5*). All amplification products were sequenced and analyzed by using the software package DNAStar (DNAStar, Madison, WI, USA). Phylogenetic trees were constructed by using the neighbor-joining method and the software program MEGA4 (www.megasoftware.net). Statistical analyses were performed by using SPSS, version 17.0 (SPSS Inc., Chicago, IL, USA).

A total of 320 (44.3%) samples were positive for rotavirus and 102 (14.1%), 27 (3.7%), and 32 (4.4%) for caliciviruses, adenoviruses, and astroviruses, respectively. A total of 17 positive samples were detected with Mon269/Mon270, and an additional 15 samples were found with primers SF0073/SF0076. Phylogenetic analysis revealed that 21 of the 32 astrovirus-positive isolates were classic HAstV, dominated by HAstV-1 (12 samples); 7 samples were AstV-MLB1(GenBank accession nos. JQ673575–JQ673581), and 4 were AstV-MLB2 or HMOAstV-A (2 isolates each) (GenBank accession nos. JQ673582–JQ673585). Primers SF0073/SF0076 detected 4 classic astroviruses that were not detected by Mon269/Mon270. We found no statistically significant difference (χ*^2^* = 1.547, p = 0.214) between the detection rates of novel astroviruses in Lanzhou and Nanjing. The prevailing astrovirus genotypes (classic and novel) in both regions were similar. Furthermore, the prevalence and genotype distribution of classic HAstV were similar to those in a previous study in China (*8*).

Rotaviruses were a co-pathogen in 14 (43.8%) astrovirus-positive fecal samples. Three samples were AstV-MLB1 positive; the remaining 11 had classic HAstV. Differences were noted between seasonality; classic astrovirus infections (66.7%) occurred during October and December, and novel astrovirus infections were observed in March, April, May, July, and November. However no statistically significant differences in mean age (p = 0.209, Student *t* test), rate of fever and vomiting (p = 0.712 and p = 0.472, respectively, Fisher exact test), or mean duration and frequency of diarrhea (p = 0.231 and p = 0.177, respectively, Student *t* test) were observed between the classic and novel astrovirus groups.

Nucleotide sequence analysis showed that the AstV-MLB1 isolates in this study had 98.64% homology, with 99.65% identity at the amino acid level in open reading frame 1b region. Further phylogenetic analysis indicated that AstV-MLB1 viruses were closely related to AstV-MLB1 HK05, with 95%–98% genomic identity, whereas AstV-MLB2 was closely clustered with the strain CRI41435, sharing 99% sequence identity. AstV-MLB1 and AstV-MLB2 are phylogenetically related to the rat astroviruses RS118 and RS126. The remaining novel astroviruses, 10322603 and 10621246, clustered closely with human, mink, and ovine astrovirus strain NI-295 (Figure).

**Figure:**
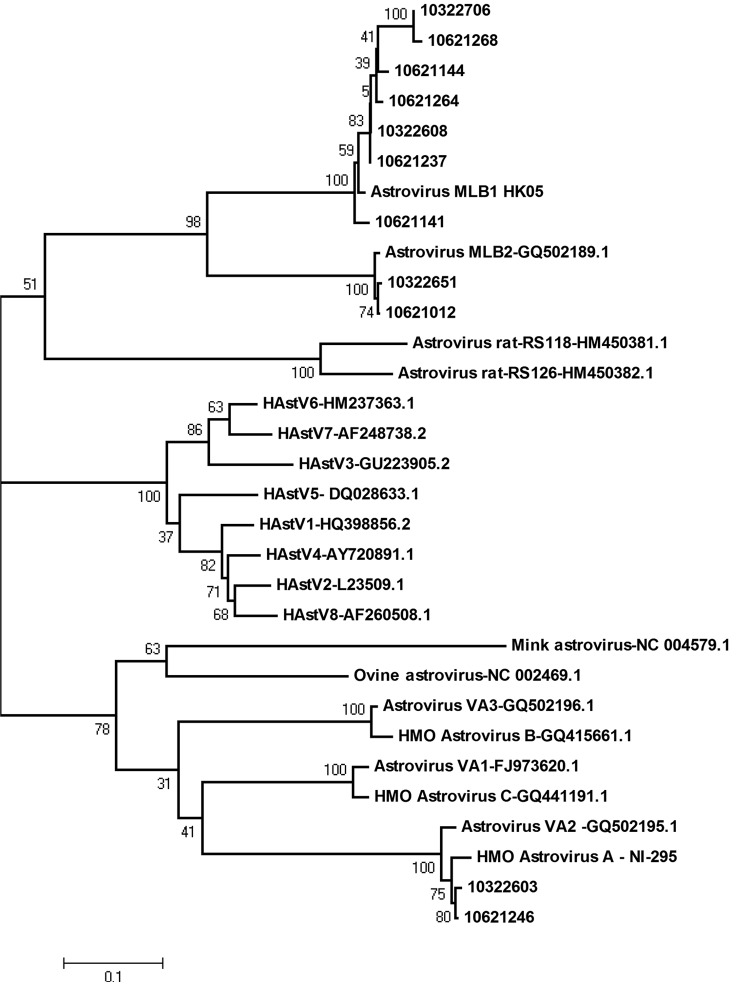
Phylogenetic analyses of human astroviruses, China. Construction of phylogenetic trees was based on alignment of a region of the open reading frame 1b nucleic acid sequence (409 bp), generated by the neighbor-joining method with 1,000 bootstrap replicates. Each strain from this study is indicated by the patient number (10621012, 10621141, 10621144, 10621237, 10621246, 10621264, 10621268, 10322603, 10322608, 10322651, 10322706) or GenBank accession number (JQ673575–JQ673585) as indicated. AstV, astrovirus; AstV-MLB, human astrovirus MLB; HAstV, human astrovirus; HMO-A, B, C, human-, mink-, and ovine-like astrovirus species A, B and C; AstV-VA, human astrovirus VA.

This study documented that multiple novel astroviruses circulated simultaneously with common human astrovirus types in China. The detection rates of novel astroviruses, especially Ast-MLB1, were higher than in 2 previous reports (*3*,*4*), although lower than in a study from Egypt (*9*). These results indicate that multiple novel astroviruses are spread worldwide. The differences in prevalence may have been caused by the geographic and/or study cohort differences. The phylogeny of astroviruses determined in our study basically agrees with previous analyses (*5*), supporting the idea that the novel astroviruses are related to other animal astroviruses. Additional studies using full-genome sequencing should be done to clarify the origin of the novel astroviruses.

One limitation of this study was that no asymptomatic control was included. A recent case–control study has suggested that AstV-MLB1 was not associated with diarrhea (*10*). However, other novel astroviruses were not assessed. Further study, especially with a large case–control cohort, should be initiated to determine the correlation of unique astroviruses with gastrointestinal and extraintestinal diseases.
